# Supplementary material for: Degradation of CdS Yellow and Orange Pigments: A Preventive Characterization of the Process through Pump–Probe, Reflectance, X-ray Diffraction, and Raman Spectroscopy
Source: Materials (Basel). 2022 Aug 11;15(16):5533. doi: 10.3390/ma15165533 (PMC9413883; doi:10.3390/ma15165533)
Supplement: Supplementary file 1 [file materials-15-05533-s001.zip › materials-1799146-supplementary.pdf]

Supporting Information

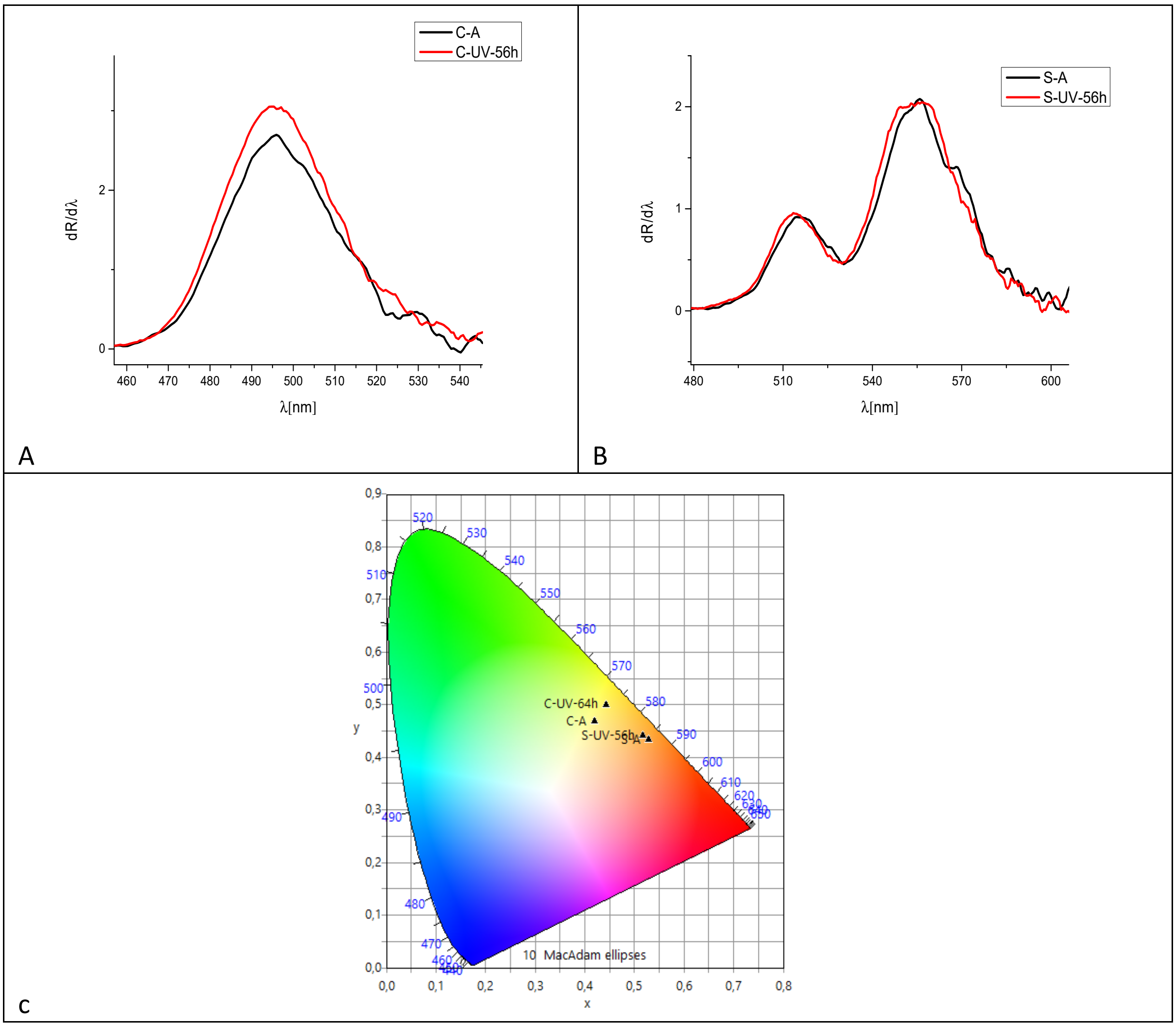

Figure S1. First derivative reflectance spectra for C-A samples (a) and S-A sample (b) before and after 56h of UV exposure, c) representation of CIE value of these samples in the CIE color space.

C-A

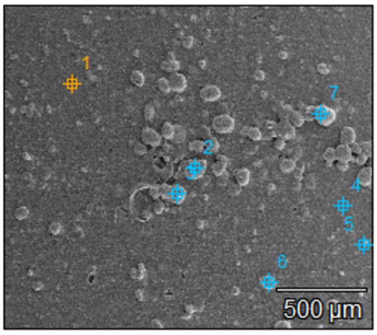

S-A

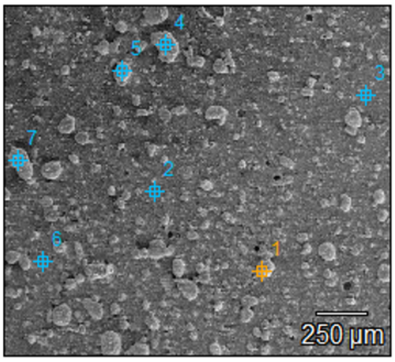

Element	pt3	_pt3	_pt3	_pt3	_pt3
	Line Type	Weight %	Weight % err	Atom %	Atom % err
O K (C)	K	18	1,4	49,9	4
S K	K	17,6	0,4	24,5	0,5
Zn K	K	4,7	0,8	3,2	0,5
Cd L	L	43,8	1,5	17,3	0,6
Ba L	L	16	1	5,2	0,3
		100		100	

Table S1. elemental compositions of C-A sample

Element	_pt7	_pt7	_pt7	_pt7	_pt7
	Line Type	Weight %	Weight % err	Atom %	Atom % err
C K (C)	K	7,4	0,6	27,5	2,1
O K (C)	K	3,4	0,6	9,5	1,6
Si K	K	7,5	0,2	11,9	0,3
S K	K	17,1	0,2	23,9	0,3
Ca K	K	1,5	0,1	1,7	0,1
Se K	K	1,7	0,3	1	0,2
Cd L	L	61,4	0,9	24,4	0,4
		100		100	

Table S2. elemental compositions of C-A sample

	C-A	S-A
$Cd_{1-x}Zn_xS$	80%	-
Cd(S,Se)	-	100%
BaSO4	20%	-

Table S3. Semi-quantitative analysis based on SEM EDX results for C-A and S-A.

In table S5 for no aged sample are presented the quantitative calculation about CdS and barium sulfate compounds, for aged sample the table shown only the calculation obtained by Cd-saturation and so the total amount 100% is calculated with respect to Cd atom%.

C-UV

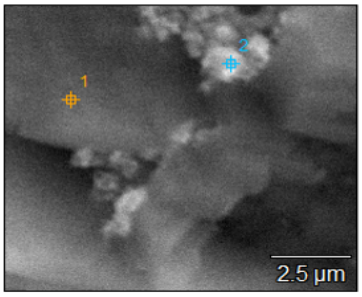

C-UV

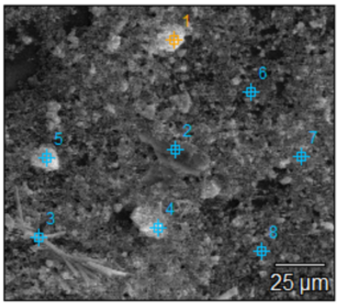

**Element**	**pt1**	**pt1**	**pt1**	**pt1**	**pt1**
	Line Type	Weight %	Weight % err	Atom %	Atom % err
**C K**	K	2,1	0,5	4,7	1,2
**O K**	K	42,2	1,3	71,2	2,2
**Al K**	K	0	0	0	0
**Si K**	K	2,1	0,2	2	0,2
**S K**	K	13,3	0,2	11,1	0,2
**K K**	K	1,1	0,2	0,8	0,1
**Ca K**	K	0,4	0,1	0,3	0,1
**Zn K**	K	3,8	0,5	1,6	0,2
**Cd L**	L	34,3	1	8,2	0,2
**Ba L**	L	0,7	0,2	0,1	0
		100		100	

**Element**	**pt2**	**pt2**	**pt2**	**pt2**	**pt2**
	Line Type	Weight %	Weight % err	Atom %	Atom % err
**O K**	K	24	1,7	55,4	3,8
**Al K**	K	0,2	0,1	0,2	0,2
**Si K**	K	2,7	0,2	3,5	0,3
**S K**	K	17,9	0,3	20,6	0,3
**K K**	K	0,9	0,2	0,9	0,2
**Ca K**	K	0,5	0,1	0,5	0,1
**Zn K**	K	5,1	0,6	2,9	0,4
**Cd L**	L	48,7	1,3	16	0,4
		100		100	

Table S4. SEM-EDX quantitative elementary analysis for sample C-UV-56h

C-500-6h

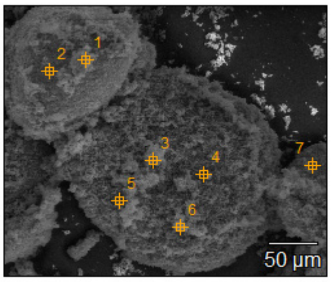

Eleme nt	pt2	pt2	pt2	pt2	pt2
	Line Type	Weight %	Weight % err	Atom %	Atom % err
**C K**	K	2,7	0,6	7,3	1,5
**O K**	K	28,9	1,5	59,7	3,2
**Mg K**	K	0,1	0,2	0,1	0,2
**Al K**	K	0,3	0,1	0,4	0,2
**Si K**	K	3,1	0,1	3,7	0,2
**S K**	K	10,8	0,3	11,1	0,3
**K K**	K	0,4	0,3	0,3	0,2
**Ca K**	K	0,3	0,1	0,3	0,1
**Zn K**	K	6,5	0,8	3,3	0,4
**Cd L**	L	46,9	1,4	13,8	0,4
		100		100	

Elemen t	pt3	pt3	pt3	pt3	pt3
	Line Type	Weight %	Weight % err	Atom %	Atom % err
**O K**	K	26,7	1,6	61,5	3,6
**Al K**	K	0,2	0,1	0,3	0,2
**Si K**	K	3,3	0,2	4,3	0,2
**S K**	K	11,3	0,3	13	0,3
**K K**	K	0,6	0,3	0,6	0,2
**Ca K**	K	0,6	0,1	0,6	0,1
**Zn K**	K	6	0,8	3,4	0,4
**Cd L**	L	44,8	1,4	14,7	0,5
**Ba L**	L	6,4	0,4	1,7	0,1
		100		100	

Table S5. SEM-EDX quantitative elementary analysis for sample C-500-6h

S-500-6h

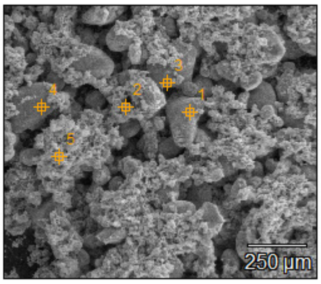

Eleme nt	pt1	pt1	pt1	pt1	pt1
	Line Type	Weigh t %	Weigh t % err	Atom %	Atom % err
**C K**	K	5,1	0,6	13,6	1,6
**O K**	K	31,2	1,5	62,3	3
**Al K**	K	0	___	0	___
**S K**	K	7,9	0,2	7,9	0,2
**K K**	K	0,8	0,2	0,6	0,2
**Cd L**	L	55	1,2	15,6	0,3
		100		100	

Eleme nt	_pt5	pt5	pt5	pt5	pt5
	Line Type	Weigh t%	Weigh t % err	Atom %	Atom % err
**C K**	K	5	0,7	13,1	1,8
**O K**	K	32	1,6	62,5	3,1
**Al K**	K	0	___	0	___
**Si K**	K	0,5	0,1	0,5	0,1
**S K**	K	8,3	0,2	8,2	0,2
**K K**	K	0,6	0,2	0,5	0,2
**Cd L**	L	52,2	0,7	15,2	0,3
		100		100	

Table S6. SEM-EDX quantitative elementary analysis for sample S-500-6h
